# Enhancing functional recovery after ACL injury. A protocol for a randomized control trial of transcranial direct current stimulation over the motor cortex

**DOI:** 10.3389/fresc.2025.1627228

**Published:** 2025-10-16

**Authors:** J. Vicente-Mampel, J. López-Soler, P. Sevilla-López, J. Ferrer-Torregrosa, J. Martín-Ruíz, E. Jaenada-Carrilero, N. Castillo-Dutor, A. Pascual-Leone, N. Pascual-Leone, L. Baraja-Vegas, A. Pascual-Leone, J. M. Tormos Muñoz

**Affiliations:** ^1^ Department of Physiotherapy, School of Medicine and Health Science, Catholic University of Valencia, Torrent, Valencia, Spain; ^2^ Department of Podiatry, School of Medicine and Health Science, Catholic University of Valencia, Torrent, Valencia, Spain; ^3^ Department of Health and Functional Assessment, Faculty of Sciences of Physical Activity and Sport, Catholic University of Valencia, Torrent, Valencia, Spain; ^4^ Department of Medicine, School of Medicine and Health Science, Catholic University of Valencia, Torrent, Valencia, Spain; ^5^ Vagelos College of Physicians and Surgeons, Columbia University, New York, NY, United States; ^6^ Hospital for Special Surgery, New York, NY, United States; ^7^ Marcus Institute for Aging Research and Wolk Center for Memory Health, Hebrew SeniorLife, Boston, MA, United States; ^8^ Department of Neurology, Harvard Medical School, Boston, MA, United States

**Keywords:** anterior cruciate ligament, motor control, exercise, neuromodulation, transcranial direct current stimulation

## Abstract

**Introduction:**

Anterior cruciate ligament (ACL) tears are common in athletic and nonathletic populations, often resulting from activities involving rapid directional changes that place stress on the knee. Although advances in surgery and rehabilitation have improved recovery, many patients still struggle to regain pre-injury performance and face increased risk of re-injury. We hypothesize that combining standard rehabilitation with transcranial direct current stimulation (tDCS) may accelerate recovery, improve neuromuscular control, and strengthen key muscles like the hamstrings and hip abductors, reducing reinjury risk.

**Methods/materials:**

This randomized controlled trial protocol, approved by the Ethics Committee of the Catholic University of Valencia, follows a double-blind, comparative, longitudinal design per SPIRIT guidelines. Elite athletes will be randomized 1:1 into two age- and sex-matched groups: non-invasive brain stimulation (NIBS) + rehabilitation (ProtocolRHB) or sham NIBS + ProtocolRHB. The NIBS intervention uses tDCS to deliver low-intensity direct current to modulate cortical excitability. Data collection spans April 2025 to December 2027 with outcomes assessed at four postsurgical time points. The primary outcome is electromyographic (EMG) activity to evaluate muscle activation, crucial for restoring knee stability and function. Secondary outcomes include knee function (Lysholm Scale) and ACL-specific quality of life. EEG and TMS will assess cortical excitability and plasticity during voluntary muscle contraction.

**Impact statement:**

This study integrates neurophysiology with rehabilitation, offering a novel approach to enhance functional recovery and lower reinjury risk post-ACL reconstruction, potentially informing future evidence-based sports medicine and neurorehabilitation strategies.

## 1 Introduction

Anterior cruciate ligament (ACL) tears are common, especially among young and active individuals (1). For people aged 10–64 years, ACL injury incidence is estimated at 0.4–0.8 per 1, 000 person-years (2–4). An estimated 65%–75% of ACL tears occur during athletic activities, including soccer, handball, skiing, and basketball, which likely accounts for injuries occurring typically in young adults aged 28–35 years old (1, 2, 4–6). Nevertheless, a significant proportion of tears, approximately 25%–35%, occur in non-athletic settings (2). Most frequently, ACL tears occur in males, who account for 58%–73% of ACL tears (2, 4, 5, 7). However, despite males engaging in more high-risk activities, when data are adjusted for exposure frequency, females are 4–8 times more prone to ACL injuries (8–13). For both males and females, proper rehabilitation is crucial for recovery which enables approximately 80% of ACL reconstruction patients to resume some form of sports activity. However, only 65% of athletes who sustain an ACL tear and undergo successful traditional ACL reconstruction return to their pre-injury performance level, and even fewer (55%) return to competitive-level physical activity. In this context, surgical approaches that go beyond simple ACL repair for more functional ACL reconstruction are important. Injury- and surgery-related complications, such as graft failure (14), muscle injuries (15), osteoarthritis, and chondral and meniscal injuries (16, 17), may indirectly impact patient rehabilitation. A critical focus of physical therapy rehabilitation intervention is to strengthen and condition critical muscles to optimize knee stabilization (18).

Leg muscles play a critical role in stabilizing the knee, particularly after an ACL tear, and thus are vital in promoting recovery and preventing re-injury. Research has shown that the hamstrings and hip abductors are essential for minimizing the risk of relapse and addressing functional shortcomings following ACL procedures (19, 20). These muscles protect the knee by counteracting ACL strain as the reconstruction heals. The contribution of muscles to ACL stress and protection varies. Muscles such as the quadriceps and gastrocnemius have shown a higher contribution to ACL stress (21). In contrast, muscles such as the hamstrings, soleus, and gluteus medius have demonstrated a significant ability to counteract ACL strain, aiding in knee stability and injury prevention. Studies using kinematics, EMG, and motor cortex outputs have expanded our understanding of the muscular involvement in ACL injuries. The voluntary activation of the quadriceps in ACL-injured individuals has been studied using measures of cortical excitability. Previous studies have used various metrics, including the active motor threshold and cortical silent period (22), and tested muscles under low-intensity electrical stimulation conditions (23). Following ACL injury, substantial neural adaptations occur within the motor system, significantly affecting voluntary muscle activation and neuromuscular control. Evidence suggests that the cortical representation of leg muscles in the primary motor cortex undergoes reorganisation after ACL, leading to altered patterns of cortical excitability and impaired motor output (24, 25). These neuroplastic changes are particularly evident in the corticomotor pathways associated with the quadriceps, where reduced force-generating capacity is linked to modifications in both intracortical and corticospinal excitabilities (26). Such adaptations are believed to be fundamental for the restoration of motor function, with bidirectional plasticity playing a critical role in the reestablishment of musculoskeletal performance after surgery (27, 28).

While most previous research has focused on the spinal-level mechanisms of arthrogenic muscle inhibition (AMI), recent findings have highlighted broader central changes. These include increased reliance on the contralateral sensorimotor cortex during movement, heightened attentional demands during proprioceptive tasks, reduced somatosensory feedback, and altered corticospinal drive (24, 29–31). Conventional physiotherapy techniques, such as standard electrostimulation and “cushion crush” strategies, have shown limited efficacy in addressing neurophysiological deficits (32). However, novel approaches, such as targeted hamstring fatigue to inhibit the flexion reflex (33), peripheral interventions such as dry needling, and the immediate integration of active rehabilitation following cryotherapy, have demonstrated promising effects in reducing AMI and improving quadriceps function (34, 35). Standard rehabilitation programs often neglect to address sensorimotor alterations and deficiencies that arise following ACL injury and reconstruction. In recent years, noninvasive brain stimulation methods have been suggested as complementary approaches to exercise, aiming to elicit central responses that enhance neuromuscular control. The application of transcranial magnetic stimulation and transcranial direct current stimulation (tDCS) to the motor cortex has shown promising results in improving motor recovery (36). tDCS works by delivering a constant, low-intensity direct current through scalp electrodes to modulate cortical excitability, while TMS uses magnetic pulses to induce electrical currents that directly stimulate neuronal firing. These techniques differ in focality and mechanism, with tDCS modulating membrane potentials and TMS producing action potentials. This study will investigate the effects of combining tDCS with exercise-based rehabilitation targeting neuromuscular control and compare this intervention with sham tDCS alongside standard rehabilitation protocols. Our primary hypothesis is that decreasing cortical hyperexcitability in the motor area, when combined with exercise, will improve neuromuscular control, leading to better outcomes across the measured parameters and a lower risk of reinjury. Additionally, it is expected that the combination of tDCS and exercise-based rehabilitation will produce superior results compared to the rehabilitation and sham tDCS intervention.

## 2 Material and methods

### 2.1 Study design

**Figure 1:**
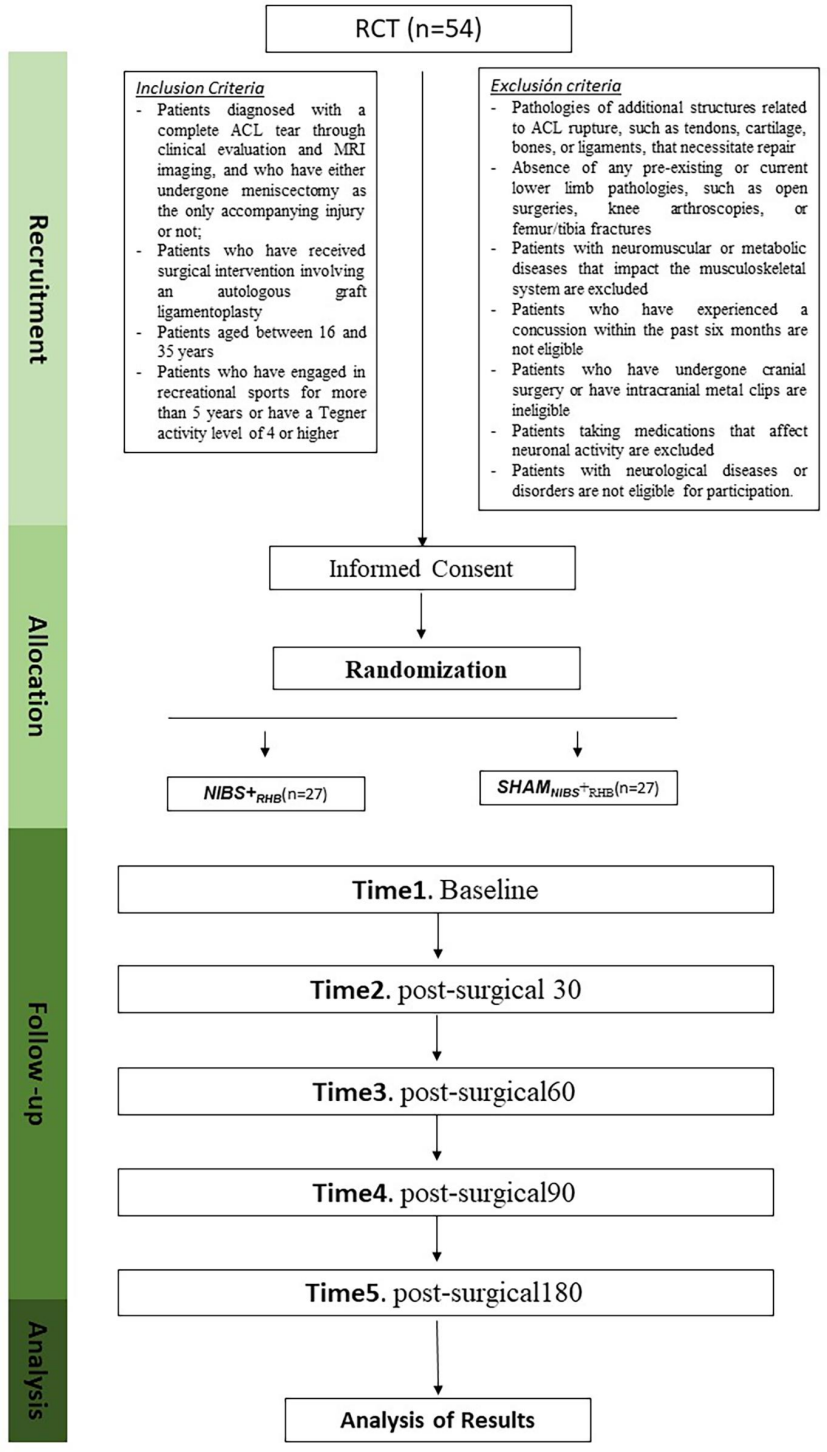
The design and progression of participants throughout the trial will be managed, including a randomization flowchart and the protocol for intervention measurements.

This study will be a randomized controlled trial featuring a double-blind, comparative, and longitudinal approach. To ensure clarity and thoroughness, the study will adhere to the SPIRIT statement guidelines (Table 1) (37). The TIDieR checklist will be used to report outcomes (38). The planned investigation will include a protocol with two comparator arms: NIBS + ProtocolRHB and ShamNIBS + ProtocolRHB, with participants randomly assigned to either arm (Figure 1). Patients will provide written informed consent prior to any study procedure, including randomization. Data collection will be conducted between April 2025 and December 2027. This study was approved by the Ethics Committee of the Catholic University of Valencia (UCV/2023-2024/053). Additionally, the study was pre-registered at https://www.Clinicaltrial.gov on 01/01/2025 (NCT06818201).

**Table 1:** Execution schedule – recruitment, intervention, and reassessment.

	Study period							
	Enrolment	Allocation	Post-allocation					Close-out
Time Point	*-t_1_*	0	*t_1_*	*t_2_*	*t_3_*	*t_4_*	*T_5_*	*t_x_*
Enrolment								
Eligibility screen	X							
Informed consent	X							
Allocation		X						
Interventions								
*[NIBS* *+* *_RHB_]*			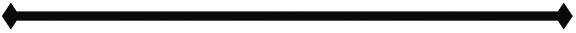					
*[SHAM_NIBS_* + _RHB_*]*			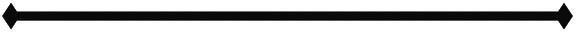					
Assessments								
*[Anthropometric data]*	X	X						
*[Psychosocial Assessment]*			X	X	X	X	X.	X
*[Scoring Scale]*			X	X	X	X	X	X
*[Functionality]*					X	X	X	X
*[EGGTMS]*			X		X		X	X
*EMGs*			X		X		X	X

t1, 2024; 0, start study; t1, postsurgical; t2, postsurgical30; t3, postsurgical60; t4, postsurgical90; t5, postsurgical180; tx, study completion.

### 2.2 Study population

Elite-level athletes will constitute the target population, with the University Clinic of the Catholic University of Valencia as the reference center. The athletes will be recruited from the national federations of handball, basketball, and rugby. Additionally, informational pamphlets will be circulated on social media platforms, allowing for additional participant recruitment. All potential participants expressing interest will receive comprehensive details about the study procedures, including the selection process.

### 2.3 Eligibility criteria

The inclusion criteria are as follows: i) individuals with a complete ACL tear diagnosed via clinical assessment and MRI scans interpreted by a board-certified radiologist; ii) patients who have undergone autologous graft ligamentoplasty; iii) individuals between 16 and 35 years of age; and iv) physically active individuals with more than 5 years of recreational sports participation. The exclusion criteria include: i) tendon, cartilage, bone, or ligament injuries requiring repair in addition to the ACL tear; ii) history of lower limb pathologies, including open surgeries, knee arthroscopies, or femur/tibia fractures; iii) presence of neuromuscular or metabolic diseases affecting the musculoskeletal system; iii) concussion within the past six months; iv) prior cranial surgery or presence of intracranial metal clips; v) use of medications affecting neuronal activity; and vi) neurological diseases or disorders.

### 2.4 Procedure

After group assignment, all participants will be assessed at four scheduled time points after surgery: one month (post-surgical30), two months (post-surgical60), three months (post-surgical90), and six months (post-surgical180). Scoring scales and psychosocial assessments will be evaluated at each visit (Figure 1). All assessments will be conducted bilaterally to allow within-subject comparisons.

### 2.5 Randomization and blinding

A block randomization design with block sizes of 4 or 6 will be implemented to ensure an even distribution of participants across groups. A double-blind design will be employed, ensuring that both the patients and the evaluators collecting data on the study variables are blinded to the adjunctive effect of complementary tDCS during the rehabilitation. To assess blinding, one might apply the recommendations of Bang et al. (39, 40). We will implement a close-ended questionnaire to enquire participants and technicians about treatment assignment with the following questions:

Question 1: “What tDCS treatment do you believe you have received/applied?” The participants/technicians will be asked to choose from three possible answers: (1) real current, (2) simulated current, or (3) do not know. If participants or therapists answered Do not know’, then Question 2 was asked: “Please provide your best guess about the tDCS treatment you received/applied and asked to choose from two possible replies”: (1) real current; (2) simulated current. Finally, we will ask for a confidence assessment with Question 3: “For the tDCS treatment you chose in question 1 or 2, please provide on a scale of 0–10 the level of confidence you have in being correct, where 0 means you are totally guessing and 10 means you are completely sure”.

### 2.6 Sample size

The required sample size was determined using GPower® software (Franz Faul, Universität Kiel, Kiel, Germany), version 3.1.9.2. A preliminary sample of 46 subjects, divided into two groups of 23 participants each, was established for the intervention design. The primary variable (EMG output) was used for statistical analysis through repeated measures ANOVA. Cohen's effect size was set at 0.357 based on a previous study examining using surface EMG recordings as their primary outcome (41). The calculation incorporated a statistical power of 0.95, an alpha level of 0.05, and two intervention groups. To account for potential dropouts during treatment follow-up (estimated at 15%), an additional 3 participants were added to each group. This resulted in a total of 54 participants, with 27 per group. The chosen effect size, exceeding 0.357, is classified as “moderate” (42). If a greater percent of participants does not follow their planned treatment protocol (dropouts, non-adherence to treatment, or missing results), an “intention-to-treat” analysis will be conducted.

### 2.7 Interventions

#### 2.7.1 NIBS_RHB_

A specialized rehabilitation protocol for postsurgical ACL recovery will be implemented. This protocol, grounded in the latest clinical guidelines (43–45), will be structured into four distinct phases: i) post-surgical recovery (weeks 0–5); ii) strength and neuromuscular control (weeks 6–12); iii) running, agility, and landings (weeks 13–24); and iv) return to sport activity (after week 24). The program will encompass 72 sessions scheduled three times a week over a 24-week period. Strength training will be the primary focus of all phases. To ensure optimal patient progress, regular assessments will be conducted with advancement to subsequent recovery phases based on the specific criteria. A schematic representation of the exercise protocol will be employed during rehabilitation (Table 2).

**Table 2:** Phased rehabilitation protocol following ACL reconstruction: objectives, progression criteria, and recommended exercises.

Phase		Weeks	Objectives	Progression criteria	Recommended exercises
Post-surgery recovery	PHASE 1.1	0–2	• Achieve fully extend the knee • Reduce inflammation • Activate the quadriceps • Full extension (0°) • Active quadriceps contraction	• Full extension (0°) • Active quadriceps contraction • Quadriceps Lag Test	• Straight leg raise (SLR): flexion, extension, abduction and adduction • Peripheral joint mobilization • Isometric quadriceps at 90°–60° • Gait training • Inflammation management: cryotherapy + compression
	PHASE 1.2	3–5		• Extension 0° • Contralateral flexion −10° • Absence of inflammation (stroke test)	Strength: • Squat (0°–60°) • Hamstring Curl • Step Up → Step Up + running technique • Lumbo-pelvic strengthening: Clamshell, Side-Lying Leg Raise Bilateral/Unilateral, Hip Hike Balance: • Progression from bilateral to single leg stance • Stationary cycling >100°
Strength and motor control	PHASE 2.1	6–8	• Restore knee strength • Restore muscle strength • Execute Single Leg Stance with proper technique	• Absence of edema • Absence of pain/edema post-exercise • Full Range of Motion (ROM) • Normal gait pattern	• Leg press, Hamstring curl, Hip abduction and adduction, Knee extension (90°–45°), Air squat with band, Deadlift • Single leg squat (SLS) progression • Progression of unilateral balance exercises to include perturbations
	PHASE 2.2	9–12		• Absence of instability episodes • 10 SLS at 60° • Functional evaluation • Quadriceps index >80% • Hamstring index >80% • Gluteus medius >80%	Previous phase + • Bilateral plyometrics in partial load • Bilateral plyometrics in full load • Submaximal training in sport-specific sagittal plane movements
Running, agility, and landings	PHASE 3.1	12–16	• Attain optimal performance in jump exercises • Complete the prescribed plyometric, agility, and running program • Regain full strength and balance capabilities	• Achieve pain-free and inflammation-free running • Functional evaluation • Quadriceps/hamstring/Gluteus medius strength >90% • Q/H RATIO >66% • Hop test >90%	Exercises (continue progressive strength training + plyometric, agility, and running block). • Bilateral sagittal plane plyometrics: broad jump, box jump, tuck jump, hop over line. • Unilateral sagittal plane plyometrics: pogo hops, lunge jump, bounding run. • Sagittal plane agility: ladder drills, forward and backward, figure-eight, deceleration, etc. • Running: adaptation protocol + running protocol (week 1)
	PHASE 3.2	16–24		• Absence of pain or inflammation • Pain-free activity performance • Appropriate movement pattern execution	Exercises (continue progressive strength training + plyometric, agility, and running block). • Frontal plane bilateral plyometrics: lateral hop over the line/hurdles, tuck jumps over the line/hurdles, etc. • Frontal plane unilateral plyometrics: lateral hop over the line/hurdles, tuck jumps over the line/hurdles, etc. • Frontal plane agility: zig-zag run, side shuffle/shuffle run, cone drills, crossover step, lateral ladder drills. • Running: running protocol (weeks 2–7).
Return to play	PHASE 4	+24	• Melbourne Return to Sport Score of 95+ • Patient demonstrates comfort, confidence, and readiness to return to sports • ACL injury prevention program implemented	• Functional evaluation • Quadriceps/hamstrings/gluteus medius strength index >90% • Q/H RATIO >70% • Hop test >90% RTP • Month 7: unrestricted training • Month 9/10 return to competition • Functional evaluation: • Quadriceps/hamstrings/gluteus medius strength index >95% • Q/H RATIO >75% • Hop test >95%	• Exercises (continue progressing strength block + plyometrics and multiple agility) • Multiple plyometrics: drop jump + rapid change of direction, 90° and 180° jumps, etc. • Multiple agility: box and start drill, lateral shuffle over the hurdles, etc.

Furthermore, the tDCS device from Ionclinics will be used in the study. The device will be applied during the early stage of rehabilitation, specifically during activation, as the exercises required will be less demanding, less intricate, and more appropriate for this intervention. The treatment plan includes 16 sessions over an 8-week period, with two sessions each week. Each session will administer a continuous current of 2 mA for 20 min. The setup comprises two electrodes (a red anode and a black cathode) and two sponge pads with conductive gel, all incorporated into a helmet tailored to the patient. Electrode placement will adhere to the international 10–20 system for tDCS (46). The primary motor cortex (M1) will be the focus of stimulation, with the anode placed at either C3 or C4, corresponding to the hemisphere contralateral to the injured leg, to target the affected motor area. The cathode will be positioned at the opposite supraorbital area, either Fp1 or Fp2. This placement ensures that stimulation is applied to the motor cortex controlling the injured limb, which is critical for modulating cortical excitability and enhancing the neuromuscular control. The tDCS stimulation will be applied prior to this early rehabilitation phase to ensure proper monitoring and to maximize the safety and efficacy of the intervention*.* The tDCS stimulation will be applied prior to this early rehabilitation phase. Specifically, during the Post-Surgery Recovery Phases 1.1 and 1.2, as well as the Strength and Motor Control Phase 2.1.

#### 2.7.2 SHAM_NIBS_ _+_ _RHB_

The device will be configured to produce an upward gradient for 30 s, identical to that used in the experimental group, followed by a downward gradient for another 30 s. Consequently, the control group will feel a tingling sensation on their scalp similar to that of the experimental group. This stimulation will occur for a total of 60 s, which is insufficient to induce changes in cortical excitability (47). Studies have demonstrated that this approach effectively ensures patient blinding (48).

### 2.8 Outcomes

#### 2.8.1 Baseline characteristics

To ensure accurate data collection for the study and monitor the patient's progress during rehabilitation, various measurements will be recorded. These will include the patient's sex, age, body mass index, athletic discipline, injury date, surgical procedure date, type of graft employed in the reconstructive surgery, time since surgery and hand/foot dominance. The study will also include measurements of knee circumference to assess volume at 5 and 10 cm above the patella's upper edge for the thigh and 5 and 10 cm below the patella's lower edge for the calf muscle. Finally, the extent of muscular arthrogenic inhibition and the patient's subjective pain perception will be evaluated.

#### 2.8.2 Primary outcome

##### 2.8.2.1 Electromyographic muscle activation

The primary outcome of this study will be the recorded electromyographic activity of eight (four on each leg) target muscles. Wireless surface electromyography (EMG) and force sensors (MuscleLab, Stathelle, Norway) connected to a 12-channel EMG amplifier (model ML6EMG01, MuscleLab, Stathelle, Norway) will be used to record muscle activity. The electrodes utilized will be the Lessa Pediatric Electrode model (30 mm diameter). The placement of electrodes will follow the SENIAM guidelines, which are part of the European Concerted Action under the BIOMED II program for noninvasive muscle assessment using surface electromyography (49). Both lower extremities will be recorded, and the placement order by channel number will be as follows: channel 1, vastus medialis (VM) (right); channel 2, rectus femoris (RF) (right); channel 3, vastus lateralis (VL) (right); channel 4, biceps femoris (BF) (right); channel 5, VM (left); channel 6, RF (left); channel 7, VL (left); and channel 8, BF (left). The muscle activity sampling rate will be 1 kHz, with each session lasting between 5 and 60 s, depending on the specific exercise.

The patients will be instructed to perform each movement with the maximum possible contraction and as quickly as possible to achieve the highest peak force (50). The patient will perform three familiarization repetitions at submaximal intensity at the beginning of each repetition (51). Assessments will first be conducted on the healthy extremity, followed by testing on the surgically repaired knee. Patients will attempt three maximum voluntary isometric contractions (MVIC), each lasting 5 s, with a 30-s rest interval between each repetition (52). A 10-min rest period will be allowed between each position to prevent the influence of fatigue on the results and to ensure optimal recovery. Additionally, the patient will receive both visual and verbal feedback (e.g., “come on”, “go ahead”) to encourage maximal effort (51).

##### 2.8.2.2 EMG analysis

Once the data are recorded, they will be stored on a hard drive in Comma-Separated Values (. csv) files. Signal analysis will be performed using MATLAB software (R2025a) (Mathworks Inc., Natick, USA). Data will be collected using a smooth-data function. Initially, a fourth-order Butterworth bandpass filter ranging from 20 to 400 Hz will be applied to process the signal. The signal will then undergo rectification or Root Mean Square (RMS) analysis by dividing the measurement section into 100 segments. Based on the exercise, we will extract segments lasting between 3 and 30 s (for analysis). The central 30 s in the resting position will be used as the baseline EMG. For dorsal flexion, plantar flexion, and the gamified approach, the central 5 s of work will be considered, and the mean RMS will be quantified for each exercise. For tests of maximum isometric contraction of knee flexion and knee extension, the central 3 s and maximum RMS will be used. In addition, the peaks of each signal will be calculated using the MATLAB “findpeaks” function, and the relationship between the signal peaks and individual muscles will be determined, which will allow the order of contraction to be established. Finally, the activation frequency of each recording will be calculated. The highest value recorded during the three trials will be selected and normalized based on each patient's body mass index to remove the influence of body mass, thereby enabling a comparison between them.

#### 2.8.3 Secondary outcome

##### 2.8.3.1 Scoring scale

###### 2.8.3.1.1 Lysholm scale

The Lysholm scale assesses knee function in different ligament injuries, with the goal of tracking progress after an intervention and/or evaluating knee deterioration under certain conditions (53). The scale consists of eight components: limping, use of support for walking, instability, pain, locking, swelling, ability to climb stairs, and ability to squat. It is rated on a scale from 0 to 100, where 100-95 is considered excellent, 94-84 is good, 83-65 is fair, and below 65 is poor. Additionally, each component and the total score are analysed separately. This scale has a Cronbach's alpha of 0.737 and an intraclass correlation coefficient of 0.844 (54).

###### 2.8.3.1.2 Anterior cruciate ligament-quality of life questionnaire

This scale is a continuous quantitative tool used as a Patient-Reported Outcome Measure to evaluate the effect of ACL injuries on patients' lives. The questionnaire comprised 32 items divided into five domains: symptoms and physical issues (five items), work-related challenges (four items), sports participation/competition (12 items), lifestyle (six items), and social and emotional factors (five items). Each domain receives a score proportional to the number of items and is assessed using a 100-millimeter visual analog scale. Higher scores reflect improved quality of life. This scale was validated in Spanish, showing a Cronbach's alpha of 0.81 and 0.94, and an intraclass correlation coefficient indicating good consistency, ranging from 0.88 to 0.96 (55).

##### 2.8.3.2 Functional assessment

Functional Jump Tests are commonly used to evaluate patients after ACL repair, especially to assess the Limb Symmetry Index (LSI) (56, 57). The current literature suggests that the normal LSI is >90% when comparing the ACL-reconstructed limb with the non-operated limb (58). Functional Jump Tests are also cost-effective and simple to assess, as they do not require extensive space or equipment, enabling the evaluation of knee functional capacity and offering a measurable metric that can be tracked over time. Ebert et al. identified eight jump tests—single hop for distance, 6 m timed hop, triple hop for distance, triple crossover hop for distance, single medial hop for distance, single lateral hop for distance, single limb countermovement jump for height, and timed speedy hop test—that were the most effective in highlighting functional limb asymmetries in patients post-ACL injury (59).

##### 2.8.3.3 Psychosocial assessment

###### 2.8.3.3.1 Fear of movement. Tampa scale of kinesiophobia

The Tampa Scale of Kinesiophobia (TSK) will be used to assess fear of movement or perceived risk of re-injury. This self-reported questionnaire consists of a series of statements, each scored on a 4-point Likert scale ranging from “strongly disagree” to “strongly agree.” Higher scores indicate greater fear of movement or re-injury, whereas lower scores suggest reduced fear levels. It is essential to address kinesiophobia early in the rehabilitation process, as it can negatively impact a patient's adherence to the prescribed rehabilitation program (60). In particular, sports health professionals should be mindful of the influence of kinesiophobia on functional assessments, as it may hinder progress and recovery (61). Research has shown that an increase in kinesiophobia is significantly associated with worse postoperative SF-36 PCS scores, highlighting its negative effect on overall physical functioning in patients after surgery (62). The TSK demonstrates excellent internal consistency, with a Cronbach's alpha of 0.90, and has a proven high test-retest reliability, with an intraclass correlation coefficient (2, 1) of 0.934 (63). Additionally, the severity of kinesiophobia following ACL reconstruction is influenced by factors such as symptom subscales and the Pain Catastrophizing Scale (PCS), further emphasizing the need to consider these factors in the rehabilitation process (64).

###### 2.8.3.3.2 Pain catastrophizing scale

The Pain Catastrophizing Scale (PCS) is a self-reported questionnaire designed to evaluate the extent of catastrophizing in response to pain in patients. It consists of 13 items, each rated on a Likert-type scale from 0 to 4, with higher scores reflecting a greater tendency to catastrophize while experiencing pain (65). The PCS is widely used to understand how individuals perceive and react to pain, as catastrophic thinking can significantly influence pain perception and coping strategies. Studies have shown that catastrophizing is a particularly influential factor in the variation of postoperative pain, with adolescents often showing more pronounced effects than adults (66). This suggests that age and developmental factors may play a role in the way catastrophizing influences pain experiences following surgery. The PCS provides a total score ranging from 0 to 52, with higher scores indicating higher levels of catastrophizing. This scale has demonstrated strong psychometric properties, including robust content and construct validity, ensuring that it effectively measures the concept it is intended to assess (67). Additionally, the PCS has been shown to exhibits excellent internal consistency and test-retest reliability, making it a reliable tool for evaluating various musculoskeletal disorders, including those related to injury and surgical intervention (68, 69). Furthermore, the PCS has been found to have significant clinical relevance, as it correlates with both pain intensity and functional outcomes, particularly in long-term recovery. For example, six months after an ACL injury, higher PCS scores were associated with increased pain levels and diminished functional ability (64). These findings highlight the importance of addressing catastrophizing in rehabilitation, as it may contribute to worse recovery. The Spanish version of the PCS has been validated and has good psychometric properties. It has an internal consistency of 0.79, which is considered acceptable, and a test-retest reliability of 0.84, indicating that the scale performs consistently over time in Spanish-speaking populations. These characteristics make the PCS a useful and reliable tool for assessing pain catastrophizing in diverse populations, ensuring that its utility extends across languages and cultures.

##### 2.8.3.4 Cortical excitability

Transcranial magnetic stimulation (TMS) is a non-invasive method developed to examine the functionality of human corticospinal pathways (70). Researchers have integrated TMS with EEG and functional imaging techniques to enhance the measurement of cortical excitability. Image guided TMS, with either population-averaged magnetic resonance images (MRIs) or patient-specific MRIs, allows for precise localization of areas of interest. In this study, Brainsight® markers will be positioned on the patient's head using the nasion and zygion as reference points for image-guidance. We will target the hand knob and assess for the motor hotspot—the area of highest motor activity in the injured and non-injured leg's quadriceps and hamstrings muscles using TMS stimulation. Using this hotspot, we will find the motor threshold, or the minimum stimulation required to elicit a response of >50uV in 5 out of 10 TMS pulses. Once the hotspot is identified and the minimum motor threshold established, a 64-channel EEG cap will be placed. EEG recordings will be conducted using a high-density TMS-compatible EEG system (BrainProducts Brain ActiChamp, Gilching, Germany) over the identified hotspot where the minimum motor threshold was obtained with the Brainsight system. The cap allows for the recording of corticospinal and EEG activity (71). For the EEG procedure, the patient will be instructed to contract quadriceps and hamstrings muscles to 30% of their maximum voluntary isometric contraction for 10 s. The patient will receive visual biofeedback through EMG during the contraction, incorporating visual gamification to enhance engagement and performance. The patient will receive visual biofeedback through EMG during the contraction, incorporating visual gamification to enhance engagement and performance. Each contraction will last a maximum of 5 s to avoid fatigue. This process will be repeated three times with intervals between each contraction (72). Latency in milliseconds, amplitude in microvolts, and pulse frequency in hertz will be used to assess cortical excitability and plasticity by recording cortical activity during voluntary contraction (73).

### 2.9 Program feasibility and safety: attendance and compliance with protocol

Several factors influence the adherence of patients with ACL injuries to their exercise regimens. Improving a patient's ability to complete a rehabilitation program, especially home exercises, can be facilitated by considering social and environmental factors that enhance adherence and compliance (74). Furthermore, following the recommended guidelines for return-to-sport clearance after ACL reconstruction is essential for effective rehabilitation (75). Key factors that impact attendance at physiotherapy appointments and participation in sessions include therapist support, rehabilitation setting, and exercise progression (76). For this study, protocol adherence will be measured by calculating the percentage of patients who complete the assessments following established methods from previous research (77). In the initial stages, individualized training will be provided to ensure treatment plan compliance and minimize the risk of adverse effects.

### 2.10 Oversight and monitoring

Specific protocols will be implemented to safeguard data and participant well-being. The principal investigators will collaborate with a physician who leads the non-invasive and precision neuromodulation institute to monitor and evaluate the study's progress and safety measures. Despite the minimal reported side effects, patients will undergo a 14-day monitoring period following the procedure. The operation will be halted if the patient reports any localized signs or symptoms of infection. The analysis of the study will utilize information gathered prior to the conclusion of the intervention. To ensure participant safety, all individuals who completed the intervention phase will receive follow-up phone calls for a week after completion. An Independent Safety Monitor, who is the ethics committee secretary that approved the study, will receive and review biannual progress reports. These reports cover participant recruitment, retention/attrition, and adverse events. At the conclusion of the study, a comprehensive report will be prepared detailing and summarizing the adverse events. The concluding report will include explanations provided by the study participants who chose to withdraw. The reasons behind their withdrawal will be examined and contrasted with the initial expectations of the researchers to uncover the patterns and possible factors influencing these decisions. Furthermore, an evaluation will be conducted to predict which participants might leave the study early, with the goal of developing strategies to mitigate this and ensure the reliability of results. The Data Safety Monitoring Plan requires that any serious adverse events be reported to the ethics committee within a 48-hour timeframe. Should an unexpected serious adverse event pose an increased risk to participants, the study will be suspended if the independent safety monitor determines it is necessary due to the frequency or severity of the events.

#### 2.10.1 Data collection

Patient medical information will be entered directly into a secure computer system located at the evaluation sites. Each patient will receive their own unique identifier to anonymize data. To enable data sharing among researchers for further analysis, an Excel file containing only unique patient identifiers will be distributed. This method safeguards the confidentiality and security of information.

#### 2.10.2 Statistical analysis

##### 2.10.2.1 Baseline characteristics

To evaluate demographic baseline measures across intervention groups, comparisons will be conducted using analysis of variance (ANOVA) or chi-square tests (i.e., NIBS + Protocol_RHB_ and ShamNIBS + Protocol_RHB_) to identify statistically significant differences between groups (*p* > 0.05).

##### 2.10.2.2 Analysis of the outcome measures

A per-protocol analysis will be performed in accordance with the CONSORT guidelines for reporting randomized controlled trials. The Kolmogorov–Smirnov test will be utilized to verify the normality assumption, and the Levene test will be used to evaluate the homogeneity of variances. To examine the effects of tDCS combined with exercise-based rehabilitation on patients undergoing ACL reconstruction, repeated measures ANOVA will be applied, with experimental groups as factors and Bonferroni corrections for *post hoc* analysis. Comparisons within and between groups for both primary and secondary outcomes will assess time, group, and interaction effects. Results will be expressed as mean differences (MD) with 95% confidence intervals (CI95%). The effect size (ES) will be calculated using Cohen's d coefficient. All statistical analyses will be conducted using SPSS 24 software (IBM Inc., Chicago, Illinois, USA). If there are participant dropouts or if the statistical power is below 80%, an intention-to-treat analysis will be implemented (78).

##### 2.10.2.3 Correlation coefficient

The strength of the relationship between the variables will be evaluated using the Pearson correlation coefficient and/or the Spearman correlation coefficient (if the normality assumption is not met).

### 2.11 Dissemination plan

A dissemination plan has been established to ensure that the study findings will be shared openly with the scientific community and other relevant stakeholders. The results will be published in peer-reviewed journals, presented at national and international conferences, and made available upon request to interested researchers. The primary objective of a dissemination plan is to ensure that research findings are effectively communicated, understood, and utilized, maximizing their potential impact. This study intends to publish its results in medical, physiotherapy, and exercise journals to make them accessible to professionals and researchers in the field. In line with open science principles, anonymized datasets and study materials will be made available in publicly accessible repositories. The key goals of the dissemination strategy will include widespread distribution of the research findings, ensuring they are clear and comprehensible to an informed audience with expertise in the field. The therapy protocols will be presented in detail to maintain transparency and facilitate replication. Findings may be shared with third parties only when justified and with the authors' consent. Through these efforts, the plan seeks to ensure that the research contributes meaningfully to both the academic community and practical applications.
