# Supplementary material for: Enhancing functional recovery after ACL injury. A protocol for a randomized control trial of transcranial direct current stimulation over the motor cortex
Source: Front Rehabil Sci. 2025 Oct 16;6:1627228. doi: 10.3389/fresc.2025.1627228 (PMC12571851; doi:10.3389/fresc.2025.1627228)
Supplement: Supplementary file 1 [file Table1.doc]

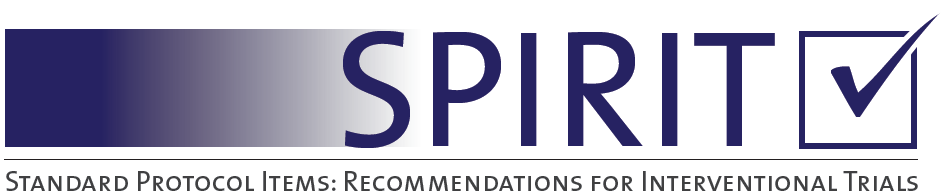


SPIRIT 2013 Checklist: Recommended items to address in a clinical trial protocol and related documents*

| Section/item | ItemNo | Description |
| --- | --- | --- |
| **Administrative information** | | |
| Title | 1 | Enhancing functional recovery after ACL injury. A Protocol for a Randomized Control Trial of Transcranial Direct Current Stimulation Over the Motor Cortex. |
| Trial registration | 2a | Trial identifier ([NCT06818201](https://clinicaltrials.gov/study/NCT06818201)). |
|  | 2b | 1. Effects of Transcranial direct current stimulation over the motor cortex during recovery of ACL Patients. A Randomized Control Trial (RCT). [NCT06818201](https://clinicaltrials.gov/study/NCT06818201).  2. Date of registration in primary registry: January, 2025  3. Secondary identifying numbers: Ethics Committee Catholic University of Valencia (protocol number UCV/2023-2024/053).  4. Sources of monetary or material support: This work was supported by Conselleria d'Innovació, Universitats, Ciència i Societat Digital Grants: The Catholic University of Valencia University and Coordinació del Sistema Valencià d'Investigació, Ciència i Desenvolupament Tecnològic Pilot Grant CIGE/2023/9 (GE).  5. Primary sponsor: Vicente-Mampel, J  6. Secondary sponsor(s): Jaenada-Carrilero E  7. Contact for public queries: juan.vicente@ucv.es ; +34  674177877; Faculty of Medicine and Health Science.  Department of Physiotherapy, Catholic University of Valencia,  Torrent, Valencia, Spain.  8. Contact for scientific queries: Not applicable.  9. Public title: Effects of Transcranial direct current stimulation over the motor cortex during recovery of ACL Patients. A Randomized Control Trial.  10. Scientific title: Effects of Transcranial direct current stimulation over the motor cortex during recovery of ACL Patients. A Randomized Control Trial  11. Countries of recruitment: Valencia, Spain.  12. Health condition(s) or problem(s) studied: Patients diagnosed  ACL Injuried  13. Intervention(s): The program will encompass 72 sessions, scheduled thrice weekly over a 24-week period. Strength training will be emphasized throughout all phases as the primary focus.  14. Key inclusion and exclusion criteria: The following criteria will determine eligibility for inclusion: i) Individuals diagnosed with a complete ACL tear through clinical assessment and MRI scans; ii) Patients who have undergone autologous graft ligamentoplasty; iii) Individuals between 16 and 35 years of age; and iv) Participants with over 5 years of recreational sports involvement or a Tegner activity level of 4 or above. Exclusion criteria encompass: i) additional pathologies related to ACL rupture requiring repair, such as tendon, cartilage, bone, or ligament issues; ii) history of lower limb pathologies, including open surgeries, knee arthroscopies, or femur/tibia fractures; iii) presence of neuromuscular or metabolic diseases affecting the musculoskeletal system; iii) concussion within the past six months; iv) prior cranial surgery or presence of intracranial metal clips; v) use of medications impacting neuronal activity; and vi) neurological diseases or disorders.  15. Study type: This study was designed a double-blinded  comparative longitudinal and prospective randomized  controlled trial (RCT).  16. Date of first enrolment: April 1, 2025  17. Target sample size: 54 participants will be recruited in total.  18. Recruitment status: Pending: Participants are not yet being  recruited or enrolled at any site.  ranges  from 0 to 5, where 5 is the highest instability level.  - Quality of life (SF-36): The SF-36 measures the quality of life and comprises  several dimensions: (a) physical functioning, (b) role physical, (c) role  emotional, (d) social functioning, (e) bodily pain and (f) vitality;  - Visual analogue scale (VAS): We will use the scale that provides values from  0 to 10. This scale is an efficient tool to quantify in a subjective and selective  way this range in which 0 is considered a total absence of pain and 10 the  worst pain imaginable.  20. Key secondary outcome(s): - Patient’s satisfaction: There are already published  studies about diagnosed patients that evaluate their satisfaction using a numeric scale  of 11 (-5 to 5) (37,38). High scores show the patient’s satisfaction with the treatment.  - Sorensen Test: Sorensen test it measures the amount of strength and  resistance of the back extensors. The subject is asked to be going prone  position against a table and to keep the trunk parallel to the floor against  gravity once the legs and pelvis are flexed. The amount of time in this position  will be measured in seconds. (39)  - Tampa Scale of Kinesiophobia: The TSK is a self-administered questionnaire  composed of different questions with a 4-point Likert scale ranging from  “strongly disagree” to “strongly agree.”  - Self-efficacy: Self-efficacy questionnaire is composed of 19 items with 3  domains that assess self-efficacy for pain management and physical  functioning.  - Pain Catastrophizing Scale: The Pain Catastrophizing Scale (PCS), a selfadministered questionnaire (13 items on a Likert-type scale from 0 to 4) was  used in this study to assess the level of catastrophizing in the presence of pain.  The total scores range from 0 to 52 points, where higher scores represent  higher levels of catastrophizing.  Protocol version 3 Date and version ident |
| Protocol version | 3 | Date and version identifier  Unique Protocol ID: UCV/2023-2024/053 Brief Title: Effects of tDCS on Motor Cortex During ACL Recovery Official Title: Effects of Transcranial Direct Current Stimulation Over Motor Cortex During Recovery of ACL Patients |
| Funding | 4 | This work was supported by Conselleria d'Innovació, Universitats, Ciència i Societat Digital Grants: The Catholic University of Valencia University and Coordinació del Sistema Valencià d'Investigació, Ciència i Desenvolupament Tecnològic Pilot Grant CIGE/2023/9 (GE). |
| Roles and responsibilities | 5a | All the authors were involved in the conception, design, and methodology of the protocol. JVM drafted the manuscript. LBV revised the manuscript for critically intellectual content. EJC and JTM reviewed and edited the manuscript. All authors read and approved the final manuscript for submission. |
|  | 5b | School of Medicine and Health Science. Department of Physiotherapy, Catholic University of Valencia, Torrent, Valencia, Spain**.** |
|  | 5c | The centers to which the researchers are affiliated intervened in the design of this study and will not have any role during its execution, analysis, and interpretation of the data, nor in the decision to present the results. |
|  | 5d | "Study Oversight Committee. Composition of the Research Ethics Catholic University of Valencia. |
| Introduction |  |  |
| Background and rationale | 6a | Anterior cruciate ligament (ACL) rupture is a common injury, particularly among young and physically active individuals, with an incidence of 0.4 to 0.8 injuries per 1,000 person-years. While the majority of ruptures occur during sports activities (65-75%), a significant proportion (25-35%) happen in non-sport settings. Rehabilitation is crucial for recovery, but only 65% of patients return to their pre-injury activity level, and only 55% resume competitive activities. Neuromuscular structures such as the hamstrings and hip abductors play a vital role in reducing the risk of re-injury and aiding post-surgical rehabilitation. Arthrogenic muscle inhibition (AMI) is a common phenomenon following ACL surgery, affecting quadriceps activation and force generation. While spinal mechanisms of AMI have been extensively studied, the influence of supraspinal centers, such as the motor cortex, in modulating AMI is also recognized. Traditional treatments, such as electrostimulation, are largely ineffective, and non-invasive brain stimulation techniques, such as transcranial direct current stimulation (tDCS), combined with exercise rehabilitation, are being explored to improve neuromuscular control. |
|  |  |  |
| Objectives | 7 | This study aims to evaluate the effects of combined tDCS and exercisebased rehabilitation, comparing it to sham tDCS treatment. Outcomes will include cortical reorganization, corticospinal activation, pain perception, and psychosocial and functional variables. The central hypothesis is that reducing cortical hyperexcitability will enhance neuromuscular control, leading to improved outcomes and a reduced risk of re-injury |
| Trial design | 8 | This study was designed a double-blinded comparative longitudinal and prospective randomized controlled trial (RCT). Once the groups have been established there will we four sample collections: After group formation, six sample collections will be conducted: Baseline, post-surgical 30 (thirty days), post-surgical60 (two months), post-surgical90 (three months) and p post-surgical180 (six months) |
| Methods: Participants, interventions, and outcomes | | |
| Study setting | 9 | The study will be performed at the Clinicas UCV of Valencia, Spain. On a sample of federated athletes will constitute the target population, with the University Clinic of the Catholic University of Valencia serving as the reference center |
| Eligibility criteria | 10 | The eligibility inclusion criteria were: i) Individuals diagnosed with a complete ACL tear through clinical assessment and MRI scans; ii) Patients who have undergone autologous graft ligamentoplasty; iii) Individuals between 16 and 35 years of age; and iv) Participants with over 5 years of recreational sports involvement or a Tegner activity level of 4 or above. |
| Interventions | 11a | A specialized rehabilitation protocol for post-surgical ACL recovery will be implemented. This protocol, grounded in the latest clinical guidelines (1–3), will be structured into four distinct phases: i) Post-surgical recovery (weeks 0-5); ii) Strength and neuromuscular control (weeks 6-12); iii) Running, agility, and landings (weeks 13-24); and iv) Return to sport activity (after week 24). The program will encompass 72 sessions, scheduled thrice weekly over a 24-week period. Strength training will be emphasized throughout all phases as the primary focus. To ensure optimal patient progress, regular assessments will be conducted, determining readiness for advancement to subsequent phases based on specific criteria. **Experimental:** The Home tDCS device (Ionclinics®) will be utilized for the tDCS intervention. This procedure will be implemented during the initial phase of rehabilitation, specifically during activation, as the required exercises will be less intense, less complex, and more suitable for this intervention. The treatment plan consists of 16 sessions spread over an 8-week period, with two sessions per week. Each session will involve a continuous current of 2 mA applied for 20 minutes. The setup includes two electrodes (a red anode and a black cathode) and two sponge pads with conductive gel, all integrated into a patient-specific helmet. Electrode placement will follow the international 10-20 system for tDCS (38). The primary motor cortex (M1) will be the target area for stimulation, with the anode positioned at either C3 or C4, and the cathode placed at the opposite supraorbital area, either Fp1 or Fp2.**Sham group.** The device will be set up to generate an upward slope for 30 seconds, matching the one utilized in the experimental cohort, followed by a downward slope for an additional 30 seconds. As a result, the control group will experience a similar tingling sensation on their scalp as the experimental group. This stimulation will last for a total of 60 seconds, which is not long enough to trigger alterations in cortical excitability (39). Research has shown that this method effectively ensures patient blinding (40). |
|  | 11b | If pain was experienced during the application of the exercise protocol, adjustments would be made to the parameters of range of motion, joint position, and exercise intensity in an effort to minimize changes to the stimulation of the target tissue |
|  | 11c | Strategies to improve adherence to intervention protocols will be exercise group should perform the therapeutic exercises following the instructed regularity according to the SIRAS scale. |
|  | 11d | It will be prohibited to perform exercise other than that established during the duration of the research. Additionally, patients will have limited the type of activities as currently presented within the established protocol. |
| Outcomes | 12 | Primary Outcome Measure: Electromyography (EMGs) The The Surface EMG amplitudes of the quadriceps and hamstring muscles. The main device, Musclelab, is equipped with a Musclelab force sensor and wireless surface elec-tromyography (EMGs). Electrode placement will follow the Surface ElectroMyoGraphy for the Non-Invasive Assessment of Muscle (SENIAM) protocol from the European Concerted Action in the Biomedical Health and Research Program (BIOMED II). The instructions provided to the patients will be to perform the movement with the max-imum possible contraction, and as rapidly as possible, to achieve the highest peak of force. The patient will carry out 3 familiarization repetitions at a submaximal inten-sity at the beginning of each repetition, and it will begin with the non-surgical ex-tremity, followed by the tests on the surgical extremity. Three effective MVIC attempts will be conducted, each lasting 5 seconds, with a 30-second rest interval between each repetition. Anterior Cruciate Ligament - Quality of Life Questionnaire (ACL - QOL) This scale is of a continuous quantitative nature. It serves as a Patient-Reported Outcome Measure to assess the impact on the lives of patients with ACL, both pre- and post-treatment, regardless of whether the treatment is surgical or non-surgical. The questionnaire comprises 32 items, categorized into 5 domains: symptoms and physical problems (5 items), work-related problems (4 items), sports participation/competition (12 items), lifestyle (6 items), and social and emotional aspects (5 items). Each domain is allocated a proportional score based on the number of items it contains and is evaluated using a 100-millimeter visual analog scale (VAS). A higher score indicates a better quali-ty of life for the patient. This scale was validated in Spanish, demonstrating a Cronbach's alpha of 0.81 and 0.94 and an intraclass correlation coefficient (ICC) that exhibited good consistency, ranging from 0.88 to 0.96. Lysholm Scale Its purpose is to evaluate knee functionality in various types of ligament injuries, with the objective of monitoring the progression following an intervention and/or assessing knee deterioration in specific pathologies. The scale comprises eight items (limping, use of support for ambulation, instability, pain, locking, swelling, ability to ascend stairs, and ability to squat). It is evaluated on a scale of 0 – 100, with 100-95 being classified as excellent, 94-84 as good, 83-65 as fair, and less than 65 as poor. Furthermore, each item and the overall score are analysed independently. This scale demonstrates a Cronbach's alpha of 0.737 and an intraclass correlation coefficient of 0.844. Functional Jump Test Functional Jump Test have been commonly used for the evaluation of patients after ACLR, especially to assess the Limb Symmetry Index (LSI). Consist in 8 jump tests: Single Hop for Distance (SHD), 6m Timed Hop (6MTH), Triple Hop for Distance (THD), Triple Crossover Hop for Distance (TCHD), Single Medial Hop for Distance (MHD), Single Lateral Hop for Distance (LHD), Single Limb Countermovement Jump for Height (SLCMJ), and Timed Speedy Hop Test (TSHT). Tampa Scale of Kinesophobia, Pain Catastrophizing Scale and EGGTMS. |
| Participant timeline | 13 | At baseline, 30days post-surgery, 60days post-surgery, 90days post-surgery and 180days post-surgery. |
| Sample size | 14 | The required sample size was determined using GPower® software (Franz Faul, Universität Kiel, Kiel, Germany), version 3.1.9.2. A preliminary sample of 45 subjects, divided into three groups of 15 participants each, was established for the intervention design. The primary variable EMGs was used for statistical analysis through repeated measures ANOVA. Based on a previous study (36), Cohen's effect size was set at 0.357. The calculation incorporated a statistical power of 0.95, an alpha level of 0.05, and three intervention groups. To account for potential dropouts during treatment follow-up (estimated at 15%), an additional 3 participants were added to each group. This resulted in a total of 54 participants, with 18 per group. The chosen effect size, exceeding 0.357, is classified as "moderate" (37). Should the final number of analyzed individuals yield a lower power than planned, an "intention-to-treat" analysis will be conducted. This approach was adopted to address potential issues such as dropouts, non-adherence to treatment, or missing results. |
| Recruitment | 15 | Federated athletes will constitute the target population, with the University Clinic of the Catholic University of Valencia serving as the reference center. Additionally, the project will be promoted through social media platforms using informational pamphlets, allowing for the evaluation of more potential subjects. Everyone will receive comprehensive details about the study procedures, and those who show interest will be given a brief overview of the selection process. |
| **Methods: Assignment of interventions (for controlled trials)** | | |
| Allocation: |  |  |
| Sequence generation | 16a | A block randomization design with block sizes of 6 or 12 will be utilized to ensure equal distribution of participants across each group. |
| Allocation concealment mechanism | 16b | Participants were randomly allocated to one of the three experimental groups for an external researcher using EPIDAT 3.1. software (<http://www.sergas.es/>). |
| Implementation | 16c | Both the participants and the researcher in charge of collecting data were unaware of the assigned intervention and the results obtained, respectively, until the end of the study. An independent researcher using an excel formula, generated a table of random numbers to blind data collectors and outcome adjudicators to ensure an unbiased outcome ascertainment |
| Blinding (masking) | 17a | The randomization sequence was saved on a USB drive and securely stored under lock and key by an independent researcher |
|  | 17b | Accessible only when absolutely necessary |
| **Methods: Data collection, management, and analysis** | | |
| Data collection methods | 18a | Instuments employed in the study have reliability and validity. Electrical activity muscle, Lishom Scale and Anterior Cruciate Ligament-Quality of Life Quiestionnaire. Single Hop for Distance, 6m Timed Hop, Triple Hop for Distance, Triple Crossover Hop for Distance, Single Medial Hop for Distance, Single Lateral Hop for Distance, Single Limb Countermovement Jump for Height, and Timed Speedy Hop Test. tests were the most suitable for demonstrating functional asymmetries of the limbs in patients after ACLR. Tampa Kinesophobia Sacale, Paon Catastophizing Sacale and Cortical Excitability with Transcranial magnetic stimulation. |
|  | 18b | Researchers must ensure clear and transparent measurement and reporting of adherence and dropout data. Moreover, individualized treatment will be applied. |
| Data management | 19 | The Data Management Plan has been meticulously designed to ensure the quality, integrity, and confidentiality of the data collected during the randomized clinical trial. The study coordinator will assume primary responsibility for data management, performing specific tasks after paper collection. Subsequently, they will create encrypted tables in Excel to ensure participant confidentiality, implementing a double-entry procedure to verify the integrity and accuracy of the records. In the context of study design, essential elements such as the number of patients, inclusion and exclusion criteria, randomization and blinding procedures, treatment and outcome variables have been clearly delineated, all documented in the methodology section of the experimental design. Data collection will be carried out through record forms and electronic systems, with a rigorous policy for eliminating incomplete data to ensure consistency at the study's end. |
| Statistical methods | 20a | Following the CONSORT guidelines for randomized controlled trial reporting, a per-protocol analysis will be implemented. The normality assumption will be checked using the Kolmogorov-Smirnov test and box-plot analysis, while variance homogeneity will be assessed through the Levene test. To assess the effects of tDCS combined with exercise-based rehabilitation on ACL reconstruction patients, a repeated measures two-way ANOVA will be employed, considering experimental groups as factors and applying Tukey corrections. Within- and between-group comparisons for primary and secondary outcomes will examine time, group, and interaction effects. Findings will be expressed as mean difference (MD) with 95% confidence intervals (CI95%). Effect size (ES) will be estimated using Cohen's d coefficient. SPSS 24 software (IBM Inc., Chicago, Illinois, USA) will be used for all statistical analyses. In the event of dropouts or statistical power falling below 80%, an intention-to-treat principal analysis will be applied (69). The statistical analysis will be conducted by a researcher not involved in data collection, who will receive coded data. |
|  | 20b | Not applicable |
|  | 20c | Not applicable |
| **Methods: Monitoring** | | |
| Data monitoring | 21a | "Study Oversight Committee. Composition of the Research Ethics Committee (CEIm) of the Catholic University of Valencia. |
|  | 21b | JVM and LBV investigators will undertake interim analyses, with exclusive access to the outcomes. This approach ensures continuous monitoring of patients and potential adverse effects. |
| Harms | 22 | Only the adverse effect of epilepsy is anticipated. This situation occurs regardless of TDCS application. |
| Auditing | 23 | The procedure and frequency for auditing the study will depend on what is established by Catholic University of Valencia, the institution responsible for the ethics committee, to ensure proper conduct of the study. |
| Ethics and dissemination | | |
| Research ethics approval | 24 | Ethical approval for this research has been granted by the Catholic University of Valencia's Ethics Committee (UCV/2023-2024/053). Furthermore, the study has been registered in advance on Clinicaltrial.gov 01/01/2025 (NCT[NCT06818201](https://clinicaltrials.gov/study/NCT06818201)).  . |
| Protocol amendments | 25 | Refinements entail establishing clear strategies or procedures to inform pertinent stakeholders about significant modifications or updates made to the study protocol. This includes continuous communication with participants, research team members, ethics committees, regulatory authorities, and providing detailed explanations of the alterations in study documents or reports throughout the research process. |
| Consent or assent | 26a | The informed consent will be obtained by the nursing team at the hospital. None of them are involved in the development of the experimental study. |
|  | 26b | Not applicable |
| Confidentiality | 27 | Nursing team at the hospital enrolled participants will be collected, shared, and maintained to protect confidentiality before, during, and after the trial. |
| Declaration of interests | 28 | The authors declare that they have no competing interests. |
| Access to data | 29 | The principal investigators will have access to the data, and they will analyze the results objectively, having declared no conflicts of interest. |
| Ancillary and post-trial care | 30 | There is no financial compensation for participation in the study. The clinical procedure implemented is the one routinely used in everyday clinical practice. |
| Dissemination policy | 31a | All results will be published in scientific journals in the fields of medicine, physiotherapy, and exercise. |
|  | 31b | All members of the research team are specialists in exercise and TDCS in ACL patients. Furthermore, they are researchers in their respective fields and therefore can contribute to the development of articles in the future publication process |
|  | 31c | The protocols implemented regarding treatments will be described to enhance transparency and increase reproducibility. Additionally, the study's results may be provided that there is a justifiable cause, and it is accepted by the authors |
| Appendices |  |  |
| Informed consent materials | 32 | Model consent is added. Appendix 1. |
| Biological specimens | 33 | Not applicable |

*It is strongly recommended that this checklist be read in conjunction with the SPIRIT 2013 Explanation & Elaboration for important clarification on the items. Amendments to the protocol should be tracked and dated. The SPIRIT checklist is copyrighted by the SPIRIT Group under the Creative Commons “[Attribution-NonCommercial-NoDerivs 3.0 Unported](http://www.creativecommons.org/licenses/by-nc-nd/3.0/)” license.
